# The History of Slavs Inferred from Complete Mitochondrial Genome Sequences

**DOI:** 10.1371/journal.pone.0054360

**Published:** 2013-01-14

**Authors:** Marta Mielnik-Sikorska, Patrycja Daca, Boris Malyarchuk, Miroslava Derenko, Katarzyna Skonieczna, Maria Perkova, Tadeusz Dobosz, Tomasz Grzybowski

**Affiliations:** 1 Department of Molecular and Forensic Genetics, Bydgoszcz, Institute of Forensic Medicine, Ludwik Rydygier Collegium Medicum, Nicolaus Copernicus University, Bydgoszcz, Poland; 2 Institute of Biological Problems of the North, Far-East Branch of the Russian Academy of Science, Magadan, Russia; 3 Department of Forensic Medicine, Wrocław Medical University, Wrocław, Poland; IPATIMUP (Institute of Molecular Pathology and Immunology of the University of Porto), Portugal

## Abstract

To shed more light on the processes leading to crystallization of a Slavic identity, we investigated variability of complete mitochondrial genomes belonging to haplogroups H5 and H6 (63 mtDNA genomes) from the populations of Eastern and Western Slavs, including new samples of Poles, Ukrainians and Czechs presented here. Molecular dating implies formation of H5 approximately 11.5–16 thousand years ago (kya) in the areas of southern Europe. Within ancient haplogroup H6, dated at around 15–28 kya, there is a subhaplogroup H6c, which probably survived the last glaciation in Europe and has undergone expansion only 3–4 kya, together with the ancestors of some European groups, including the Slavs, because H6c has been detected in Czechs, Poles and Slovaks. Detailed analysis of complete mtDNAs allowed us to identify a number of lineages that seem specific for Central and Eastern Europe (H5a1f, H5a2, H5a1r, H5a1s, H5b4, H5e1a, H5u1, some subbranches of H5a1a and H6a1a9). Some of them could possibly be traced back to at least **∼**4 kya, which indicates that some of the ancestors of today's Slavs (Poles, Czechs, Slovaks, Ukrainians and Russians) inhabited areas of Central and Eastern Europe much earlier than it was estimated on the basis of archaeological and historical data. We also sequenced entire mitochondrial genomes of several non-European lineages (A, C, D, G, L) found in contemporary populations of Poland and Ukraine. The analysis of these haplogroups confirms the presence of Siberian (C5c1, A8a1) and Ashkenazi-specific (L2a1l2a) mtDNA lineages in Slavic populations. Moreover, we were able to pinpoint some lineages which could possibly reflect the relatively recent contacts of Slavs with nomadic Altaic peoples (C4a1a, G2a, D5a2a1a1).

## Introduction

The Slavs are the most numerous population of Central and Eastern Europe. Nowadays, about 268 million people inhabiting well over half of Europe speak thirteen languages (and their dialects) belonging to the Slavic language group [1]. First remarks about Slavic populations can be found in the written sources of East Roman and Byzantine authors, dated around 550 AD. Although these reports characterized Slavic tribes that had lived at that time, the antecedent history of that ethnic groups remained unrevealed. Therefore, the issue of the earliest location of Slavs has been deliberated over the past century from the historical, archaeological, ethnographic and linguistic viewpoint (e.g. [1]–[5].) Although the ongoing discussion in these fields unanimously indicates their common origin, the results of various studies indicate different time and place of their formation. A number of alternative “homelands” of the Slavs have been suggested thus far (reviewed briefly in [1]). Many hypotheses have been proposed to explain the origin of Slavs, but the most widely discussed are “autochthonous” and “allochthonous” (migrationist) theories [6]. The “autochthonous” conception points to the continuity of cultural development of the Central Europe (the area between the Oder and Vistula rivers) from the Bronze Age until the historical appearance of the Slavs in the early Middle Ages. According to this hypothesis, the Bronze and Iron Age Lusatian culture of the area between the Oder and Vistula is considered as Proto-Slavic and subsequent Zarubinets-Przeworsk cultures (200 BC –200 AD) are included in the putative Slavic “homeland” [7], [8]. From the very beginning, this hypothesis has been challenged by some German archeologists who claim that the Przeworsk region was more arguably within an early Germanic-speaking territory [2]. Alternatively, the “allochthonous” (migrationist) assumption tracks the earliest Slavic territory to the area stretching from Carpathian foothills to the Pripet and the left bank of the middle Dniepr river (the area of contemporary Ukraine). According to this concept, the early Slavic culture assembled during the 5^th^ century AD in the area of Cherniakhovo culture and then expanded very rapidly across Central and Eastern Europe due to extensive migrations [9]. To some extent, these two contradictory views on the earliest location of the Slavs have been verified with the recent findings of physical anthropology. Based on the newest anthropological data it has been suggested that the area between the Oder and Vistula rivers witnessed continuity of human settlement between the Roman period and the early Middle Ages. Indeed, based on morphological features of skeletal materials it has been established that populations of the Przeworsk, Wielbark and Cherniakhovo cultures from the Roman period bear close similarities to the early medieval Western Slavs and not to the medieval Germanic-speaking populations [10], [11]. Furthermore, paleodemographic studies also point to the biological continuity of the populations inhabiting the Oder and Vistula basin in the Roman period and the early medieval Slavic populations of this region [10]. Therefore, anthropological data received thus far make the “allochtonic” hypothesis less plausible, especially in its extreme migrationist form.

Since an analysis of maternal mtDNA lineages appears to be an informative approach in the reconstruction of the past demographic events [12], mtDNA population studies could in principle shed more light into the complex processes leading to crystallization of a Slavic identity. However, the formation of contemporary European ethnic groups (including the Slavs) is a relatively recent process. Therefore, mtDNA analyses of lower resolution, including research into control region sequences alone, did not allow identification any specific features which would clearly distinguish Slavs from the other European populations [13]–[23]. Thus, only the studies on large amount of complete mitochondrial genome sequences may point out mtDNA lineages, which could have been involved in the formation of Slavs. According to the published data, a detailed phylogeny of relatively young branches of haplogroup H seems to have a pivotal role in exploration of the recent settlement processes in Europe [12]. In this respect, the studies performed so far focused mainly on two haplogroups H1 and H3, observed with the highest frequencies in European populations [24]–[26]. The third most common in Europe and the least studied is haplogroup H5. This subclade reaches maximal frequencies in Southern Europe (4–6%), but is virtually absent in the Middle East [25], [27]–[29]. In Slavic populations, H5 is observed at frequencies from 3.2% in Ukraine to 2.5% in Poland, Russia, Czech Republic and Slovakia ([14]–[23] and data presented in this study). Due to the higher occurrence of H5 in southern European populations and evolutionary age of ca. 16 ky, which coincides with the period of increased migration from southern to northern parts of Europe after the LGM, the reconstruction of its H5 phylogeny could provide an important information on relatively recent processes that took place in the history of European continent. It is also worth noting that some clusters of H5, like H5a could have been of central European origin [14], [24], [27], [30], [31]. Thus, one may assume that phylogenetic reconstruction of H5 together with the information on the geographical distribution of haplotypes belonging to this clade could be helpful in evaluating the origin of Slavs.

Haplogroup H6 is also very interesting because two its branches, H6a and H6b, are characteristic, correspondingly, for European and Central Asian/Near Eastern populations, thus reflecting a long-time separation of Asian and European H6 mtDNA pools [27]. Meanwhile, H6a is relatively frequent in some Slavic populations (such as eastern Slovaks [32]), but its phylogenetic structure on complete mtDNA level is poorly studied yet.

Therefore, we have determined 63 entire mitochondrial genome sequences belonging to haplogroups H5 and H6 identified in Slavic populations and reconstructed here their detailed, phylogeographic picture, accompanied by coalescence age estimates of the nodes of interest in terms of Slavic history.

Previous studies revealed the presence of mtDNA haplotypes representing non-European mtDNA lineages among Poles, Russians, Czechs and Slovaks [23], [30], [32], [33]. The occurrence of these components in the mtDNA pool of Slavs was probably of two origins: some of them point to relatively recent (medieval) contacts between Slavic and non-Slavic populations [30], [32], but some of them testify to earlier contacts (as early as 10 kya) occurred in European prehistory [33]–[35]. Thus, in order to deepen the understanding of complex Slavic history and unravel their interactions with other ethnic groups, we demonstrate here the results of complete mitochondrial genome characterization of several non-European lineages (East Eurasian A, C, D, G and African L haplogroups) found in populations of Poles and Ukrainians.

## Materials and Methods

### Ethic Statement

The study was approved by the Bioethics Committee of the Ludwik Rydygier Collegium Medicum, Nicolaus Copernicus University in Bydgoszcz, Poland (statements no. KB/32/2002, KB/299/2003, KB/414/2008 and KB/466/2010). Written informed consent from all participants was obtained before sample collection and subsequent analysis.

### Mitochondrial DNA Sequencing

Buccal swabs or blood samples were collected from maternally unrelated 404 Poles, 159 Ukrainians and 85 Czechs. Of the Polish samples 203 were collected in the northern part of Poland, in the region called Kaszuby (Kashubia), and a significant part of this population consists of descendants of the Pomeranian branch of Slavs called Kashubians. The remaining 201 samples were collected in the most southern part of Poland, in the Podhale located in the Carpathian mountains. Czech samples were randomly collected in different parts of the Czech Republic. Ukrainian samples were originated from the western part of Ukraine (Lviv region).

DNA was isolated with standard phenol/chloroform extraction. Hypervariable segments I and II (HVSI and HVSII) sequencing was performed according to Malyarchuk et al. [15]. Subhaplogroup status of haplotypes assigned into haplogroup H (223 samples) was determined on the basis of coding region SNPs analysis using the methodology proposed by Brandstätter et al. [36]. Whole mitochondrial genome sequencing was performed for 63 samples belonging to haplogroups H5 (n = 51) and H6 (n = 12) (Table S3). These samples were selected out of about 2700 samples (including 648 samples presented here) of Eastern (Russians and Ukrainians) and Western Slavs (Czechs, Poles and Slovaks) that had been screened previously for haplogroup-diagnostic coding region markers and subjected to control region sequencing [14], [15], [16], [17], [23], [30], [32]. Moreover, entire mitochondrial genome sequencing was performed for samples collected from Poles and Ukrainians, classified into haplogroups A (n = 1), C (n = 3), D (n = 1), G (n = 2) and L (n = 3) (Table S3). Complete genome sequencing was performed as described by Torroni et al. [37]. All mtDNA sequences were compared with the revised Cambridge Reference Sequence (rCRS) [38] using the SeqScape v. 2.5 software (Applied Biosystems). The GenBank accession numbers for the complete mitochondrial genomes reported in this paper are JX128041-JX128091, JX266260-JX266269 and JX307099-JX307110.

### Data Analysis

Phylogenetic reconstruction of haplogroups H5, H6, A, C, D, G and L was performed on the basis of mitochondrial genome sequences presented herein as well as FamilyTree project and previously published data (Table S1). Updated haplogroup nomenclature follows that proposed by van Oven and Kayser [39].

The most parsimonious trees of the complete sequences were reconstructed manually and verified with the Network 4.5.1.2 program (Fluxus Engineering) and mtPhyl software (http://eltsov.org). The haplogroup divergence estimates and their error ranges were calculated using mtPhyl software (http://eltsov.org), according to calibration methods proposed by Mishmar et al. [40], Kivisild et al. [41] and Soares et al. [42]. Coalescence ages estimated using the rho statistic were verified by maximum likelihood (ML) estimates of branch lengths using PhyML v 3.0 software [43], assuming HKY85 mutation model. The obtained values were converted to time using molecular clock of Soares et al. [42]. Point indels and transversions located between nucleotide positions (nps) 303–315 and 16180–16193 were disregarded during phylogenetic analysis.

## Results and Discussion

### Mitochondrial Control Region Sequences of Slavic Populations

The results of sequence variation and haplogroup assignment of 404 Poles, 157 Ukrainians and 85 Czechs are presented in Table S2. 635 haplotypes were classified into West Eurasian haplogroups (H, HV, V, J, T, U, I, W, X), seven into East Asian haplogroups A (1 sample), C (3 samples), D (1 sample), G (2 samples) and three into African haplogroup L (Table 1). As expected, the most frequent haplogroup in populations studied is haplogroup H that encompasses 42% in average. Between-population comparisons for distribution of all mtDNA haplogroups and subhaplogroups demonstrate that only haplogroup HV0* (as non-V sequences defined by 16298-72 diagnostic mutations in the mtDNA control region) was revealed more frequently in Ukrainians (P = 0.002; Fisher’s exact test) and haplogroup H6 was more frequent (P<0.02) both in Ukrainians (8.7%) and Czechs (2.5%) than in Poles (0.2%). Comparison with previously published data on Slovak mtDNA variation indicates that eastern Slovaks are also characterized by higher frequency of H6 (4.4%) [32].

**Table 1:** Frequencies of the major mtDNA haplogroups in Poles, Ukrainians and Czechs.

Haplogroup	Poles (Kashubia)	Poles (Podhale)	Ukrainians	Czechs
A	0	1	1	0
C	1	0	2	0
D	0	1	0	0
G	1	0	1	0
H*	18	20	15	6
H1	24	24	19	13
H2a	5	6	2	2
H3	1	4	4	2
H4	6	4	3	2
H5	4	5	4	2
H6	1	0	4	7
H7	3	8	4	1
H8	1	1	0	0
H10	8	3	1	0
H11	6	5	3	4
H13a	1	2	1	2
H15	0	1	0	1
HV*	3	1	3	1
HV0*	1	0	7	1
I	4	4	4	1
J*	1	1	1	0
J1	25	13	10	8
J2	2	2	2	4
K	6	9	8	5
L	1	2	0	0
N1b,c	0	4	0	0
T*	12	3	1	0
T1	4	5	3	2
T2	11	10	10	8
U1a'c	2	2	0	0
U2e	2	1	3	2
U3	2	4	2	1
U4*	0	2	0	0
U4a	8	6	7	1
U4c	0	0	0	2
U5a	11	22	15	3
U5b	11	10	5	1
U7	1	0	0	1
U8	1	2	1	0
V	6	7	4	2
W	5	5	5	0
X	4	1	2	0
In total	203	201	157	85

### Phylogenetic Reconstruction of the European Haplogorup H5 Subclades Observed in Slavic Populations

Although the investigation into mitochondrial DNA control region variation in European populations showed no significant differences between Slavs and other Europeans [14]–[16], [23], analysis of the entire genome variability of samples belonging to haplogroup H5 showed significant diversity in the structure and time of origin of its younger subbranches. The detailed phylogeny of haplogorup H5 is presented in Figure S1. The majority of complete mtDNA sequences included in the H5 phylogenetic tree are of European origin, some of which coming from individuals of no certain ethnicity (sequences determined by Howell et al. [44], Coble et al. [45], Behar et al. [46] and obtained in the framework of Family Tree DNA Project [47]). Nevertheless, a few samples from the Middle East and Africa could also be observed. The topology of the H5 haplogroup tree reflects its relatively large amount of internal variation. Within this clade, we were able to identify several new branches. In particular, new subhaplogroup H5u1 defined by motif 5899.1C-10595-12855-16400 is present in three individuals of Russian origin, and new subbranch H5x defined by a transition at the control-region position 16129 was found in one Italian sample and two samples of unknown European origin (Figure S1). We have also identified a number of new subclades represented by two haplotypes. The vast majority of them (H5a1a1, H5a1a2, H5a1a3, H5a1r, H5a1s, H5a1t, H5a6b, H5a8, H5a9, H5b4, H5e1a1, H5w, H5y, H5z, H5aa, H5ab, H5v1, H5r3, H5e1b, H5b5, H5b6, H5a7a, H5a7b, H5a6b, H5a2a, H5a1u, H5a1w, H5a3a1a) are defined by mutations at highly evolutionary stable mtDNA positions that could be observed in the global phylogenetic tree only incidentally. The only exceptions are subhaplogroups H5v with a defining mutation occurring at a position 709 and H5x subclade with a defining transition at a position 16129 (Figure S1).

**Figure 1:**
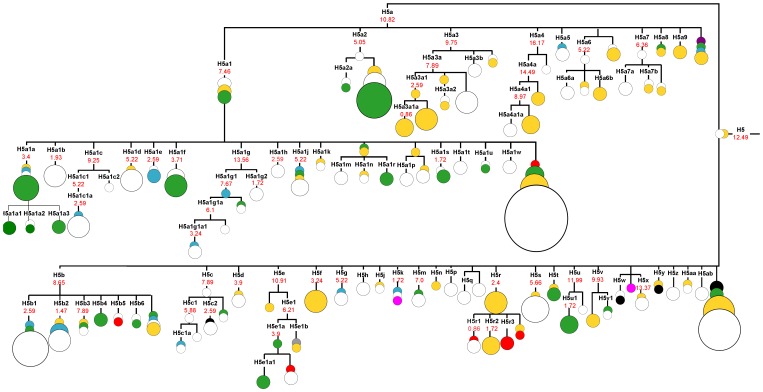
Complete mtDNA phylogenetic tree of haplogroup H5. The schematic tree is based on phylogenetic tree presented on Figure S1. Time estimates (in kya) shown for mtDNA subclades are based on the complete genome substitutions [42]. The circle size is proportional to the number of individuals sharing the haplotype. Geographical origin is indicated by different colours: green - central and eastern Europe (Czech Republic, Poland, Russia, Slovakia, Ukraine); yellow – southern Europe (Italy, Spain); red – western Europe (Austria, Germany, Netherlands); blue – northern Europe (Denmark, Finland, Ireland, United Kingdom, Orkney Islands); violet – Africa (Tunisia); black – Near East (Israel, Jordan); pink – southern Caucasus (Armenia, Georgia); grey – America (Philadelphia); white - unknown origin.

The phylogeographic picture of the entire mitochondrial genome sequences belonging to haplogroup H5 is illustrated in Figure 1. The founding node of H5 haplogroup is represented by one Italian individual and one sample of unknown ethnic origin. The H5 haplogroup shows a coalescence time of about 11.5–16.5 ky, depending on the mutational rate values. It is worth noting that the oldest subbranches within this haplogroup are H5a3, H5a4 and H5e, which are dated to 9.5–17, 16–21.5 and 11–18 ky, respectively (Figure S1), are mainly represented by the samples from southern Europe (Italy). This picture implies formation of haplogroup H5 during pre-Neolithic times, at the end of last glacial period in the areas of southern Europe. Thus, the presented results may provide further evidence of resettlement of Europe from southern European refugia after Last Glacial Maximum (LGM) [12], [48]–[50]. More detailed analysis of the topology of the haplogroup H5 tree allows us to point out the lineages that seem specific for Central and Eastern Europe (H5a1f, H5a2, H5a1r, H5a1s, H5b4, H5e1a, H5u1 and subbranches of H5a1a). The branches predominantly represented by Slavic haplotypes are certainly much younger than those principally composed of samples of southern European origin, and could possibly be traced back to 4 kya. In particular, haplogroup H5a2 is almost exclusively represented by Slavs (Poles, Russians, Slovaks and Ukrainians) and is dated at 2.5–5 ky. Furthermore, H5e1a branch comprising of one founder haplotype of Polish origin, two Russian samples, one German sample and two haplotypes of unknown origin could be traced back to 2.5–5 ky. H5u1 subclade, represented exclusively by three samples from the European part of Russia and dated to 1.7 ky also seems predominantly Slavic. In respect to these results one may note that the formation of several H5 subbranches of putative Slavic origin coincides with the time and place of origin of previously described haplogroup U4a2, which is predominantly found in Central and Eastern Europe and dates to ∼6–7 kya. U4a2 haplogroup is divided into three subclusters U4a2a, U4a2b and U4a2c that spread almost exclusively among Slavs (Poles, Russians, Ukrainians and Slovaks) [51]. It is also worth noting that two subbranches of previously described U5a2 subhaplogroup: U5a2a and U5a2b1, which are frequently observed among Poles, Russians, Belarusians and Czechs are dated to ∼6–7 kya [52]. This probably reflects distribution of the Chalcolithic and Early Bronze Age Corded Ware European cultures, as it has been suggested earlier on the basis of phylogeographic distribution of Y-chromosome R1a1a1-M458 subcluster characterized by similar expansion time [53]. Taken together, the time of origin and territorial range of mitochondrial subhaplogroups H5a2, H5e1a, H5u1, U4a2, U5a2a and U5a2b1 observed in central and eastern European populations indicate that some of the maternal ancestors of today's Slavs (Poles, Czechs, Slovaks, Ukrainians and Russians) inhabited areas of Central and Eastern Europe much earlier than it was estimated on the basis of archaeological and historical data. Indeed, we show here the existence of genetic continuity of several maternal lineages in Central Europe from the times of Bronze and Iron Ages. Thus, the data from complete mitochondrial genomes collected so far seems to indicate that the ancestors of Slavs were autochthonous peoples of Central and Eastern Europe rather than early medieval invaders emerging in restricted areas of the Prut and Dniestr basin and expanding suddenly due to migration, as suggested by some archeologists [9]. In this respect, the complete genome data on several mitochondrial subhaplogroups of probable Central European origin presented in this and previous studies [51], [52] are in a perfect agreement with the recent findings of physical anthropology, suggesting continuity of human settlement in central Europe between the Roman period and the early Middle Ages [11] as well as with earlier anthropological data pointing to the central Europe as the “homeland” of Slavs [54].

### Phylogenetic Reconstruction of Haplogorup H6 Subclades Observed in Slavic Populations

Phylogenetic analysis of 58 complete mitochondrial genomes, including 12 mtDNAs sequenced in our study (Table S3), has shown that, in accordance with phylogenetic tree proposed by van Oven and Kayser [39], haplogroup H6 comprises 3 subclades – H6a, H6b and H6c. H6a is typical to European populations, while H6b is the most frequent in Central Asia and Near East [26], [27]. Subhaplogroup H6c defined by mutations at nps 6869 and 9804 (“H9” in study [27]) is very rare, being found only eight times in individuals of European descent (Figure S2). In our study H6c was detected three times – in Czechs, Poles and Slovaks. Lack of H6c-diagnostic mutations in the mtDNA control region does not allow us to estimate the geographic pattern of distribution of this subhaplogroup. However, there is a branch of H6c defined by HVS1 motif 16362–16400, enabling to somewhat clarify this issue. Population screening has shown that this H6c-branch has been rarely revealed among Slovaks (0.5% [32]), Slovenians (0.8% [31]), Bulgarians (0.1% [55]), Ukrainians (1.3%, present study) and Lithuanians (0.6% [56]).

**Figure 2:**
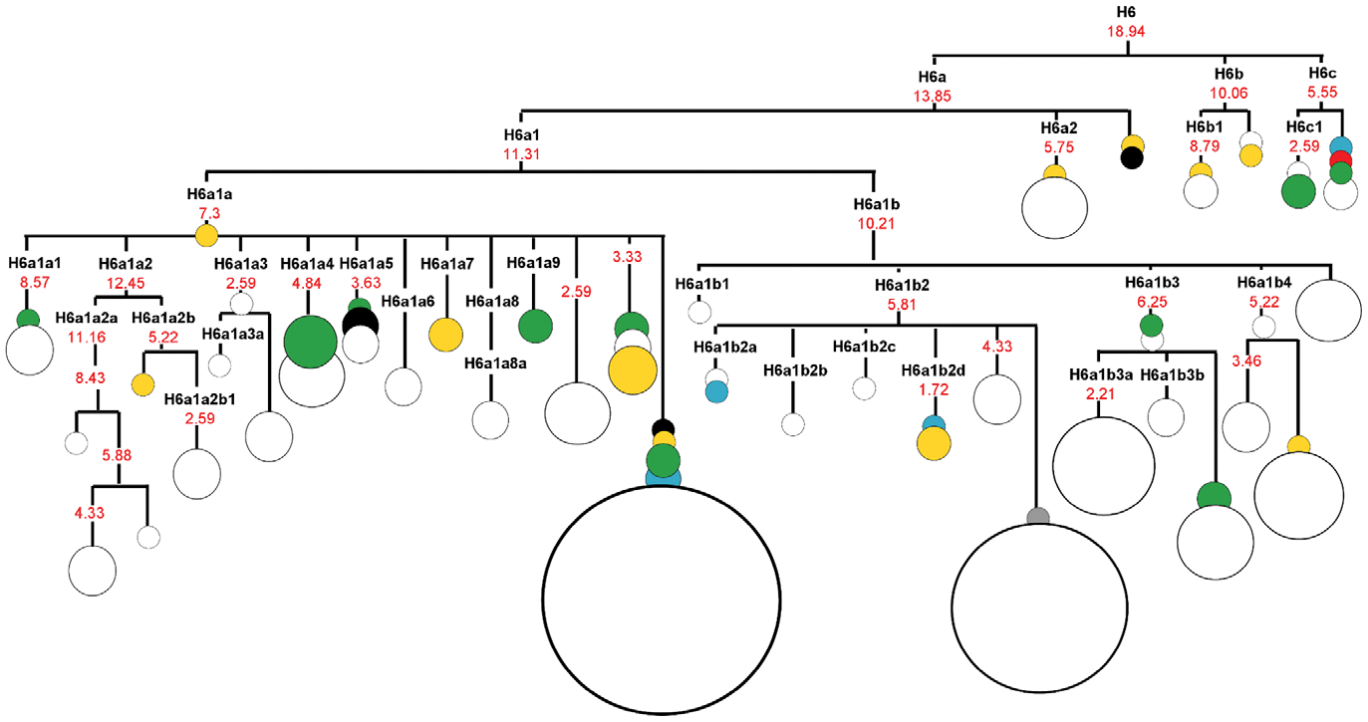
Complete mtDNA phylogenetic tree of haplogroup H6. The schematic tree is based on phylogenetic tree presented on Figure S2. Time estimates (in kya) shown for mtDNA subclades are based on the complete genome substitutions [42]. The circle size is proportional to the number of individuals sharing the haplotype. Geographical origin is indicated by different colours: green - central and eastern Europe (Czech Republic, Poland, Russia, Slovakia, Ukraine); yellow – southern Europe (Italy, Spain); red – western Europe (Austria, Germany, Netherlands); blue – northern Europe (Denmark, Finland, Ireland, United Kingdom, Orkney Islands); black – Near East (Israel, Jordan); grey – America (Philadelphia); white - unknown origin.

The phylogeographic picture of haplotypes belonging to haplogroup H6 is shown in Figure 2. Haplogroup H6 appears to be very old being dated around 15–33.5 kya, while H6a demonstrates the coalescence time of 12.5–19 kya. Subhaplogroup H6c, albeit it shows young age (of 5–9 kya), appears to be fairly ancient, because H6-phylogeny suggests a deep split between ancestors of subclusters within haplogroup H6 occurred in the pre-LGM time. It is possible that H6c-subcluster survived the last glaciation in one of the refuges (probably in the Balkans or the Ukraine) and has undergone expansion only in the last millennium, together with the ancestors of some European groups, including the Slavs.

The remaining H6 samples sequenced in our study belong to different H6a subclusters being identified as singletons (H6a1a*) or as members of subclusters H6a1a4, H6a1a9 and H6a1b3. Subcluster H6a1a9 is novel, comprising of two haplotypes found in Russians and Ukrainians. Subcluster H6a1b3 is also interesting because it contains, except for European individuals of unknown origin, a founder haplotype of Czech origin and two Polish haplotypes.

### East Asian Mitochondrial DNA Lineages in Slavic Populations

**Figure 3:**
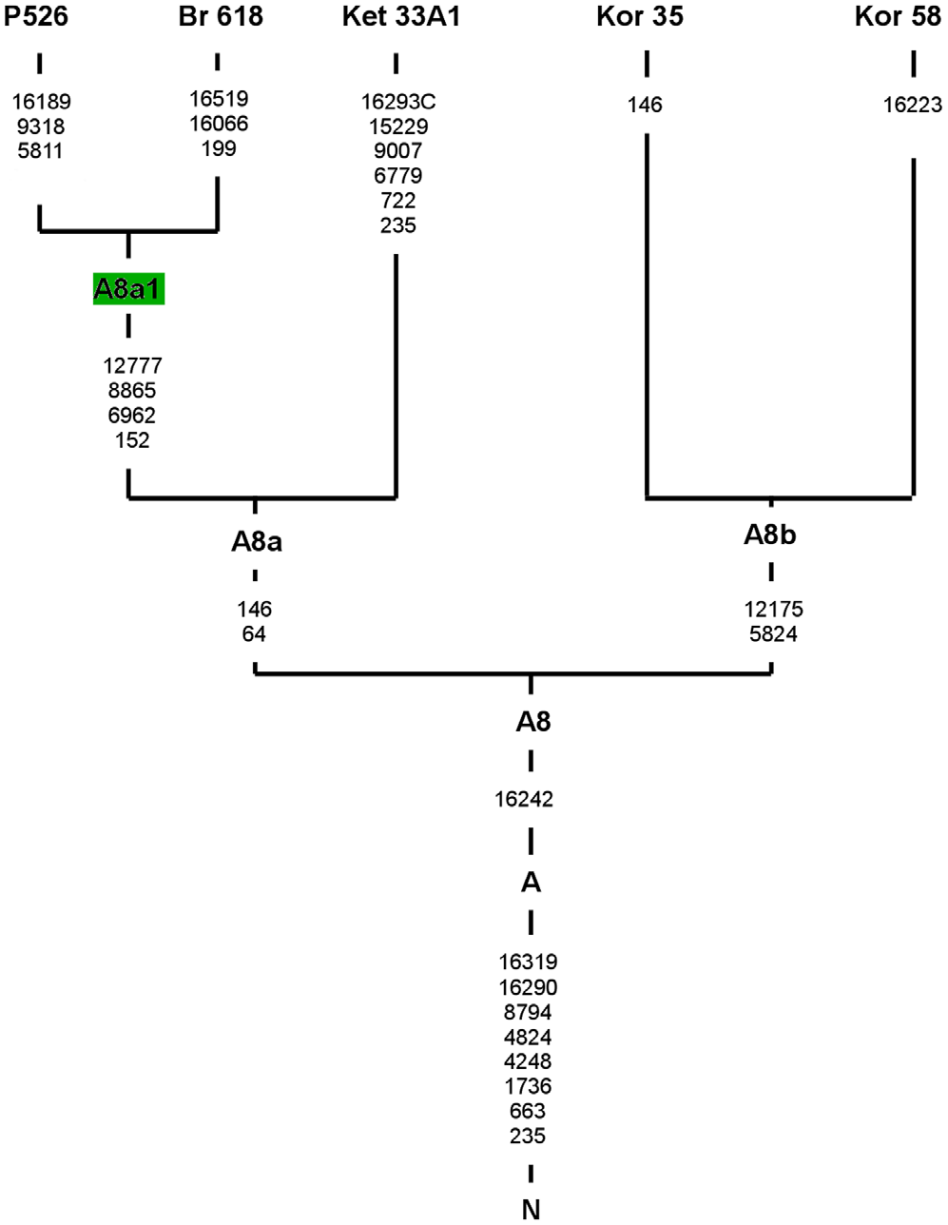
Complete mtDNA phylogenetic tree of subhaplogroup A8. The tree is rooted in haplogroup N. Mutations are scored relative to rCRS. Four additional complete genome sequences were taken from the literature listed in Table S1. The mutations are transitions, unless suffix (A, C, T or G) that represents the transversion is added. Insertions are marked by an “ins” and deletions are marked by “del” following the position of inserted or deleted nucleotide. Haplogroup names are in boldface. Haplotype names are in green. The mutations that are haplogroup diagnostic are listed below the haplogroup name. Branches consisting haplotypes identified in Slavic populations are highlighted in green.

Haplogroup A is observed with the highest frequency in eastern and northern Asia. Among 11 subhaplogroups identified so far within this clade [39], the most numerous and the best characterized are A4 and A5 subbranches. The least characterized thus far is subhaplogroup A8, the updated phylogeny of which is presented in Figure 3. A8 is defined by a transition at the control region position 16242 and is found with relatively low frequencies in populations of central and western Siberia and in Volga-Ural region [57]–[59]. Entire mitochondrial genome sequences of this clade derived from the literature come from four individuals of Asian origin. Two of those are Koryak samples that locate at the same A8b branch defined by transitions at highly conserved positions 5824 and 12175 (Figure 3). Two others (Buryat and Ket), together with one sample from Polish population determined in this study, share two mutations at highly variable positions 64 and 146 and form the second subclade (A8a). Within A8a, Polish and Buryat haplotypes are clustered together into subhaplogroup A8a1, defined by 152-6962-8865-12777 motif (Figure 3). The presence of the A8a1 haplotype among Poles supports the previous hypothesis that occurrence of A8a lineages in central Europe may reflect the probable medieval migration of the nomadic tribes from Siberia [58].

**Figure 4:**
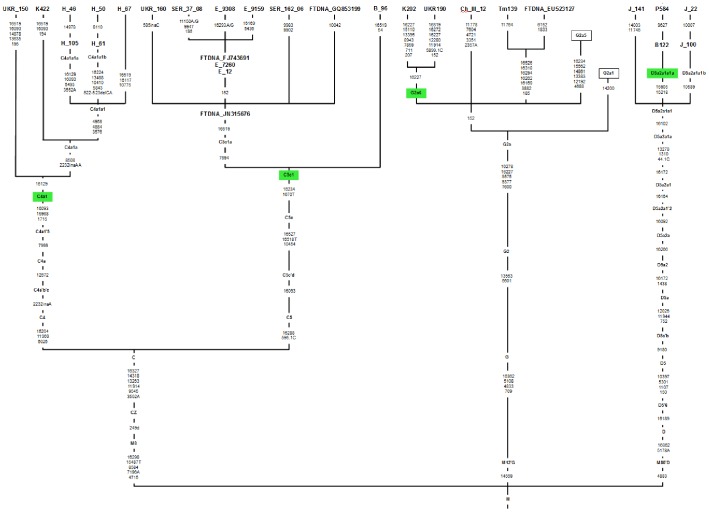
Complete mtDNA phylogenetic tree of subhaplogroups C, D, G. The tree is rooted in haplogroup M. Mutations are scored relative to rCRS. The mutations are transitions, unless suffix (A, C, T or G) that represents the transversion is added. Insertions are marked by an “ins” and deletions are marked by “del” following the position of inserted or deleted nucleotide. Haplogroup names are in boldface. Haplotype names are in green. The mutations that are haplogroup diagnostic are listed below the haplogroup name. Complete mitochondrial genome sequences taken from the literature are listed in Table S1. Branches consisting haplotypes identified in Slavic populations are highlighted in green.

The phylogeny of Asian haplogroups C, D and G is shown in Figure 4. Haplogroup C is present with the highest frequency in the populations of central Asia, while its incidence decreases in the areas of southeastern Asia and India. In the populations of Central and Eastern Europe, frequency of haplogroup C is very low [34]. Nevertheless, some subclusters of haplogroup C, like C5c1 clade was reported to occur almost exclusively in Europe [34]. C5c1 haplogroup was previously observed in three Poles [34], four Europeans [46], one Russian [47], one Caucasian and one person of unknown origin [34], [46], [47]. In this study we present an additional haplotype belonging to C5c1 clade, which was found in the Ukrainian mtDNA pool (Figure 4). As the components of the C5c1 haplogroup are virtually absent in Asia and were reported only in the populations of Central Europe, the previous hypothesis by Derenko et al. [34] that the C5c1 clade might be a marker of Siberian ancestry in Central European populations could be further supported by the results of this study. Thus, the presence of C5c1 clade among recent Europeans may reflect their ancient contacts with Asian populations, that could be traced back to the Neolithic period, as the evolutionary age of C5c1 clade was calculated to around 4–9 kya (Table S4).

Detailed analysis of the complete mitochondrial genome sequences belonging to the haplogroup C in Poles and Ukrainians show the existence of representatives of the clade C4a1, previously unobserved in other European populations. On the basis of 16129 transition, Polish and Ukrainian samples locate at the same branch as haplotypes recently found in populations of India [59]. Moreover, sample from Poland, together with Indian samples could be further classified into haplogroup C4a1a defined by 2232.2A-8508 motif. In contrast to mtDNA lineages derived from the East India, which can be grouped together on the basis of 4958-4884-3576 motif, sample from Poland determined in this study locate on a separate branch (Figure 4). Similar picture could also be observed in the case of Slavic haplotypes belonging to haplogroups G2a and D5a2a1a1. The G2a clade defined by 7600-9377-9575-16227-16278 motif is mainly represented by samples from eastern India and China. Majority of these haplotypes form G2a1 branch defined by a transition at position 14200. The G2a1 subhaplogroup was shown to be a sister clade of Polish and Ukrainian haplotypes that localize on separate branch G2a6, defined by 16227 transition (Figure 4). The topology of reconstructed haplogroup D5a2a1a1 phylogeny is more complex. The D5a2a1a1 clade, defined by a transition at a position 16102, distinguishes three main branches. Two sister clades are represented by Japanese haplotypes, while the remaining branch is formed by two individuals form Poland. On the basis of 15218–15905 motif, both Polish haplotypes form a new subhaplogroup D5a2a1a1a identified in this study. Although two Polish haplotypes locate near the sister clade composed of Japanese samples, the entire haplogroup D5a2 was seen to be represented by the sequences from India, China and South Siberia (Buryatia). The observed close proximity of six Slavic sequences belonging to clades C4a1a, G2a and D5a2a1a1 with samples of Indian and East Asian ancestry seems unexpected. Nevertheless, several reports indicate that these mitochondrial lineages are characterized by a very wide geographical distribution that covers areas of northern, eastern and central Asia [60], [61]. The occurrence of Asian haplotypes belonging to C4a1a and G2a clades among European populations probably reflects a complex interaction between these two populations, that could took place during Upper Paleolithic period, as C4a1 and G2a haplogroups could be dated back to 18–25 ky (Table S4). On the other hand, it is worth to note that C4 and D5 mitochondrial lineages are characterized by a very wide geographical distribution that covers areas of northern, eastern and central Asia [60], [61]. Thus it was proposed that these haplogroups might spread from southern China to India as a result of consequential expansion [59]–[61]. Subsequent migration through East Asia to Europe might have brought some of these components into Central Europe. Therefore, in respect to haplotypes from D5a2a1a1 clade we could not exclude the possibility that their presence in European population might be associated with relatively recent medieval events that took place in Europe. According to archaeological data, during Middle Ages, Asian populations, including Altaic tribes (Huns, Avars, and Mongols) were engaged in wars on the European continent [4], which could in consequence leave their traces in the form of Asian haplotypes in European populations.

### Haplogroup L Subclades in mtDNA Pools of Slavic Populations

**Figure 5:**
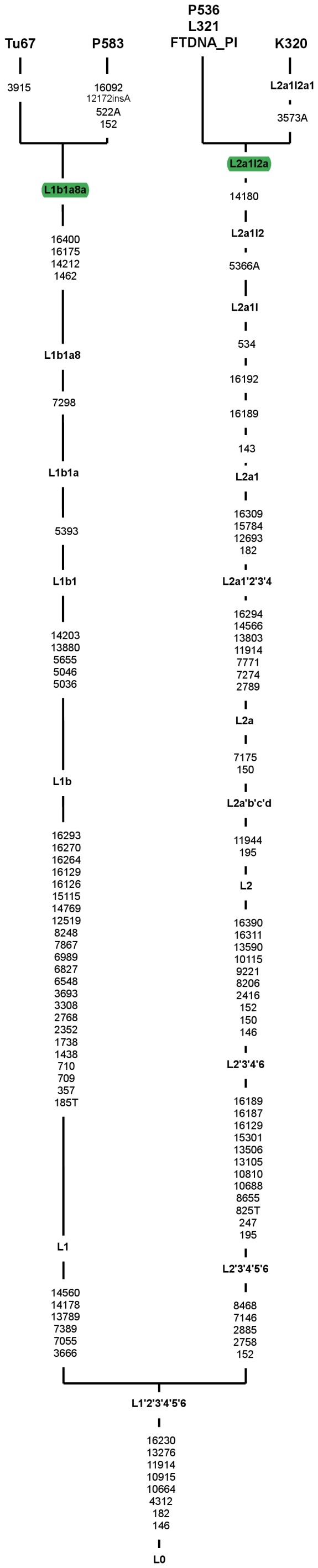
Complete mtDNA phylogenetic tree of subhaplogroups L. The tree is rooted in haplogroup L0. Mutations are scored relative to rCRS. The mutations are transitions, unless suffix (A, C, T or G) that represents the transversion is added. Insertions are marked by an “ins” and deletions are marked by “del” following the position of inserted or deleted nucleotide. Haplogroup names are in boldface. Haplotype names are in green. The mutations that are haplogroup diagnostic are listed below the haplogroup name. Complete mitochondrial genome sequences taken from the literature are listed in Table S1. Branches consisting haplotypes identified in Slavic populations are highlighted in green.

It was shown that some haplogroup L subclusters are found at low frequencies (less than 1%) in European populations [62]. The most common African clade in Europe is haplogorup L1b [63]. Recent studies carried out by Cerezo et al. [64] showed several new branches of haplogroup L (L1b1a6a, L1b1a8, L1b1a9a, L1b1a11, L1b1a14) that are mainly represented by European samples. Haplogroup L1b1a8, defined by transition at a position 7298 (not observed in the non-European L lineages) is represented by three Andalusians, one Galician [64] and one Russian [33]. In this study we identified another haplotype belonging to this clade in Polish population. Despite of the fact that L1b lineages are of West African origin [33], [64], the L1b1a8 clade probably arose *in situ* in Europe, since L1b1a8 defining mutation was observed so far in L1b clade among European haplotypes only [33], [64]. It is worth noting that Polish L1b1a8 haplotype together with Russian sample form a new L1b1a8a subcluster, defined by 1462-7298-14212-16175-16400 motif (Figure 5).

Haplogorup L2a is widespread within Africa [62]. Two representatives of the L2a clade were found in this study among Polish individuals. Both of them could be assigned to the L2a1l2a clade and locate on the same branch with the Ashkenazi Jewish haplotype (Figure 5) and Polish sample determined in a Family Tree Project. It was shown on the basis of control region variability that many of the L2a1 haplotypes are observed in Germany, Romania, France, Russia and Poland. As L2a haplotypes were reported in Ashkenazi Jewish population [65], [66], the samples representing haplogroup L2a1l2a might be further evidence of the occurrence of Ashkenazi Jewish mtDNAs in Polish mtDNA pool. It is worth noting that several Ashkenazi-specific haplotypes, belonging to clade K1a1b1a had already been found in Poles and Polish Roma. As speculated previously, Ashkenazi-specific haplotypes might have been introduced into Polish mtDNA pool about 300–400 years ago during the migration wave from Germany to Poland [23].

### Conclusions

In order to deepen the understanding of the origin of the Slavs, we have completely sequenced the mtDNAs of 63 Slavic samples representing haplogroups H5 and H6. Comparison of these haplotypes with the available complete mtDNA sequences allows us to identify a number of novel subclades. Further analysis enables us to demonstrate that both the founder node and the oldest subclusters within haplogroup H5 could be traced back to the time of last glacial period or even earlier in the case of haplogroup H6. These are mainly represented by samples of southern European origin, which further supports the idea of Europe repopulation from southern European refugia after LGM [12], [46], [47]. As expected, we show here that potentially Slavic-specific components of H5 haplogroup are much younger than H5 subclades of southern Europe, as their evolutionary age was calculated to approximately 4 kya. The formation of these clades coincides with the expansion of Central and Eastern European haplogroups U4a2, U5a2a and U5a2b1 [48], [49]. Taken together, this data points to a genetic continuity of several maternal lineages in Central Europe from the times of Bronze and Iron Ages. Interestingly, this picture could be also confirmed by expansion time of Y-chromosome subcluster R1a1a1-M458 [51]. Thus, one may exclude the migrationist assumption that Central European territories were populated by the Slavs only at the very beginning of sixth century, following whole scale depopulation of the northern areas of Central Europe [1]. Indeed, the data presented herein indicates that visible changes of material culture of Central Europe in the fifth century did not result from extensive demographic changes, but were rather accompanied by continuity of some maternal and paternal lineages between Bronze and early Middle Ages.

On the other hand, analysis of entire mitochondrial genomes of Asian and African lineages (A, C, D, G, and L) found in Poles and Ukrainians allows us to reconstruct relatively recent events in the history of Slavs, in terms of their relationships with other ethnic groups. The results presented here provide an additional evidence for the existence of limited maternal gene flow between East Asia and Central Europe. In particular, among Poles we show the presence of A8a1 haplotype, which may reflect the probable medieval migration of the nomadic tribes from Siberia. On the other hand, the existence of haplotypes representing clades C4a1a, G2a and D5a2a1a1 in Polish and Ukrainian populations could further reflect the influx of Asian haplotypes during the Middle Ages wars, in which Altaic tribes were engaged. Furthermore, in the present study we show Ashekanazi-specific L2a1l2a lineage in Polish population, which in addition to K1a1b1a haplotypes previously identified in Poland [23], constitutes further evidence of relatively recent migration of Ashkenazi Jews from Germany to Poland.

## Supporting Information

Figure S1 **Phylogenetic tree of haplogroup H5 created with mtPhyl program.** Haplogroup names are in boldface. The mutations that are haplogroup diagnostic are listed above the haplogroup name. The mutations are transitions, unless suffix (A, C, T or G) that represents the transversion is added. Insertions are marked by an “ins” and deletions are marked by “del” following the position of inserted or deleted nucleotide. Haplotype names are in green. Sequences taken from GenBank are listed in Table S1. Time estimetes (in kya) calculated according to Mishmar et al. [40] (in red), Kivisild et al. [41] (in green) and Soares et al. [42] (in blue) are shown below haplogroup names.
(XLSX)

Figure S2 **Phylogenetic tree of haplogroup H6 created with mtPhyl program.** Haplogroup names are in boldface. The mutations that are haplogroup diagnostic are listed above the haplogroup name. The mutations are transitions, unless suffix (A, C, T or G) that represents the transversion is added. Insertions are marked by an “ins” and deletions are marked by “del” following the position of inserted or deleted nucleotide. Haplotype names are in green. Sequences taken from GenBank are listed in Table S1. Time estimates (in kya) calculated according to Mishmar et al. [40] (in red), Kivisild et al. [41] (in green) and Soares et al. [42] (in blue) are shown below haplogroup names.
(XLSX)

Table S1 **List of mtDNA haplotypes retrieved from GenBank.**
(XLS)

Table S2 **Control region sequences and haplogroup assignment of haplotypes from Poland (Podhale, Kaszuby), Ukraine and Czech Republic.**
(XLSX)

Table S3 **Ethnic origin of the samples for which complete mitochondrial genome sequences were determined in the current study.**
(XLSX)

Table S4 **Age estimates (in kya) calculated according to Mishmar et al.** [**40**] **, Kivisild et al.** [**41**] **and Soares et al.** [**42**] **for haplogroups H5, H6, C4a1a, C5c1 and G2a.**
(XLS)
